# Supplementary material for: Clinical Relevance of TP53 Mutation and Its Characteristics in Breast Cancer with Long-Term Follow-Up Date
Source: Cancers (Basel). 2024 Nov 21;16(23):3899. doi: 10.3390/cancers16233899 (PMC11640694; doi:10.3390/cancers16233899)

Table S1. Patients' characteristics based on *TP53* mutation type within the *TP53*-mutated group

	Missense mutation (N=96)	Other mutations (N=76)	*p*-value
Age, median [IQR]	52 [34—76]	52 [27—87]	0.383
Histologic subtype			0.201 †
Ductal	79 (82.3)	69 (90.8)	
Lobular	2 (2.1)	0	
Others and Mixed	15 (15.6)	7 (9.2)	
Histologic grade			0.495
Grade III	57 (59.4)	49 (64.5)	
Grade I-II	39 (40.6)	27 (35.5)	
HR status #			0.425
Positive	36 (37.9)	24 (32.0)	
Negative	59 (62.1)	51 (68.0)	
HER2 status			0.625
Positive	44 (45.8)	32 (42.1)	
Negative	52 (54.2)	44 (57.9)	
Molecular subtype #			0.568
HR-positive/HER2-negative	18 (18.9)	12 (16.0)	
HER2-positive	44 (46.3)	31 (41.3)	
Triple-negative	33 (34.7)	32 (42.7)	
LVI #			0.943
Positive	33 (34.7)	26 (34.2)	
Negative	62 (65.3)	50 (65.8)	
Ki67 index (cutoff 20%)			0.175
High	67 (69.8)	60 (78.9)	
Low	29 (30.2)	16 (21.1)	
Neoadjuvant chemotherapy			0.757 †
Yes	7 (7.3)	4 (5.3)	
No	89 (92.7)	72 (94.7)	
T stage *			0.026 †
T1	45 (50.6)	24 (33.3)	
T2	43 (48.3)	43 (59.7)	
T3-4	1 (1.1)	5 (6.9)	
N stage *			0.303
N0	52 (59.8)	45 (65.2)	
N1	30 (34.5)	17 (24.6)	
N2-3	5 (5.7)	7 (10.1)	
Breast operation			0.003
BCS	51 (53.1)	23 (30.3)	
Mastectomy	45 (46.9)	53 (69.7)	
Axilla surgery			0.628 †
No approach	3 (3.1)	3 (3.9)	
SLNB	78 (81.3)	57 (75.0)	
ALND	15 (15.6)	16 (21.1)	
Adjuvant chemotherapy *			0.11
Yes	74 (83.1)	66 (91.7)	
No	15 (16.9)	6 (8.3)	
Post-operative radiotherapy			0.022
Yes	61 (63.5)	35 (46.1)	
No	35 (36.5)	41 (53.9)	

# Patients for whom accurate test values could not be confirmed were excluded.

* Patients who received neoadjuvant chemotherapy or did not undergo surgery were excluded.

† Fisher's exact test was performed.

Abbreviations, IQR; inter-quartile range, HR, hormone receptor, HER2; human epidermal growth factor receptor 2, LVI; lymphovascular invasion, BCS; breast-conserving surgery, SLNB; sentinel lymph node biopsy, ALND; axillary lymph node dissection.

Table S2. Patients' characteristics based on location of *TP53* mutation within the *TP53*-mutated group

	DNA-binding domain (N=151)	Other locations (N=21)	*p*-value
Age, median [IQR]	54 [27—87]	52 [37—75]	0.383
Histologic subtype			0.14 †
Ductal	127 (84.1)	21(100)	
Lobular	2 (1.3)	0	
Others and Mixed	22 (14.6)	0	
Histologic grade			0.352
Grade III	95 (62.9)	11 (52.4)	
Grade I-II	56 (37.1)	10 (47.6)	
HR status #			0.014
Positive	48 (32.0)	12 (60.0)	
Negative	102 (68.0)	8 (40.0)	
HER2 status			0.081
Positive	63 (41.7)	13 (61.9)	
Negative	88 (58.3)	8 (38.1)	
Molecular subtype #			0.018
HR-positive/HER2-negative	24 (16.0)	6 (30.0)	
HER2-positive	63 (42.0)	12 (60.0)	
Triple-negative	63 (42.0)	2 (10.0)	
LVI #			0.542
Positive	53 (35.3)	6 (28.6)	
Negative	97 (64.7)	15 (71.4)	
Ki67 index (cutoff 20%)			0.063
High	115 (76.2)	12 (57.1)	
Low	36 (23.8)	9 (42.9)	
Neoadjuvant chemotherapy			> 0.999 †
Yes	10 (6.6)	1 (4.8)	
No	141 (93.4)	20 (95.2)	
T stage *			0.736
T1	62 (44.0)	7 (35.0)	
T2	74 (52.5)	12 (60.0)	
T3-4	5 (3.5)	1 (5.0)	
N stage *			0.313
N0	87 (64.0)	10 (50.0)	
N1	40 (29.4)	7 (35.0)	
N2-3	9 (6.6)	3 (15.0)	
Breast operation			0.058
BCS	69 (45.7)	5 (23.8)	
Mastectomy	82 (54.3)	16 (76.2)	
Axilla surgery			> 0.999 †
No approach	6 (4.0)	0	
SLNB	118 (78.1)	17 (81.0)	
ALND	27 (17.9)	4 (19.0)	
Adjuvant chemotherapy *			0.078 †
Yes	120 (85.1)	20 (100)	
No	21 (14.9)	0	
Post-operative radiotherapy			0.42
Yes	86 (57.0)	10 (47.6)	
No	65 (43.0)	11 (52.4)	

# Patients for whom accurate test values could not be confirmed were excluded.
* Patients who received neoadjuvant chemotherapy or did not undergo surgery were excluded.
† Fisher's exact test was performed.
Abbreviations, IQR; inter-quartile range, HR, hormone receptor, HER2; human epidermal growth factor receptor 2, LVI; lymphovascular invasion, BCS; breast-conserving surgery, SLNB; sentinel lymph node biopsy, ALND; axillary lymph node dissection.

Table S3. Patients' characteristics between missense hotspot mutations and other mutations within the *TP53*-mutated group

	Missense hotspot mutations (N=39)	Other mutations (N=133)	*p*-value
Age, median [IQR]	53 [34—76]	53 [27—87]	0.953
Histologic subtype			0.176 †
Ductal	37 (94.9)	111 (83.5)	
Lobular	0	2 (1.5)	
Others and Mixed	2 (5.1)	20 (15.0)	
Histologic grade			0.99
Grade III	24 (61.5)	82 (61.7)	
Grade I-II	15 (38.5)	51 (38.3)	
HR status #			0.77
Positive	13 (33.3)	47 (35.9)	
Negative	26 (66.7)	84 (64.1)	
HER2 status			0.236
Positive	14 (35.9)	62 (46.6)	
Negative	25 (64.1)	71 (53.4)	
Molecular subtype #			0.448
HR-positive/HER2-negative	7 (17.9)	23 (17.6)	
HER2-positive	14 (35.9)	61 (46.6)	
Triple-negative	18 (46.2)	47 (35.9)	
LVI #			0.018
Positive	7 (18.4)	52 (39.1)	
Negative	31 (81.6)	81 (60.9)	
Ki67 index (cutoff 20%)			0.361
High	31 (79.5)	96 (72.2)	
Low	8 (20.5)	37 (27.8)	
Neoadjuvant chemotherapy			0.273 †
Yes	4 (10.3)	7 (5.3)	
No	35 (89.7)	126 (94.7)	
T stage *			0.002 †
T1	24 (68.6)	45 (35.7)	
T2	10 (28.6)	76 (60.3)	
T3-4	1 (2.9)	5 (4.0)	
N stage *			0.085
N0	25 (75.8)	72 (58.5)	
N1	8 (24.2)	39 (31.7)	
N2-3	0	12 (9.8)	
Breast operation			< 0.001
BCS	26 (66.7)	48 (36.1)	
Mastectomy	13 (33.3)	85 (63.9)	
Axilla surgery			0.098 †
No approach	3 (7.7)	3 (2.3)	
SLNB	32 (82.1)	103 (77.4)	
ALND	4 (10.3)	27 (20.3)	
Adjuvant chemotherapy *			0.168 †
Yes	28 (80.0)	112 (88.9)	
No	7 (20.0)	14 (11.1)	
Post-operative radiotherapy			0.003
Yes	30 (76.9)	66 (49.6)	
No	9 (23.1)	67 (50.4)	

# Patients for whom accurate test values could not be confirmed were excluded.

* Patients who received neoadjuvant chemotherapy or did not undergo surgery were excluded.

† Fisher's exact test was performed.

Abbreviations, IQR; inter-quartile range, HR, hormone receptor, HER2; human epidermal growth factor receptor 2, LVI; lymphovascular invasion, BCS; breast-conserving surgery, SLNB; sentinel lymph node biopsy, ALND; axillary lymph node dissection.

Figure S1. Kaplan-Meier curve for (A) LRFS, (B) RRFS, and (C) DMFS in patients stratified by *TP53* mutation. Stratified log-rank test and Cox regression analysis presented that there were no significant differences between the two groups. (A) The 5-year LRFS rates: 96.7% (95% CIs, 92.3–98.6) in the *TP53* mutation group vs. 97.9% (95% CIs, 95.9–98.9) in the *TP53* wild-type group; the 10-year LRFS rates: 93.6% (95% CIs, 87.2–96.8) in the *TP53* mutation group vs. 96.9% (95% CIs, 94.6–98.3) in the *TP53* wild-type group (HR, 1.82; 95% CIs, 0.76–4.38; $p = 0.173$). (B) The 5-year RRFS rates: 99.4% (95% CIs, 95.7–99.9) in the *TP53* mutation group vs. 99.3% (95% CIs, 97.9–99.8) in the *TP53* wild-type group; the 10-year RRFS rates: 97.5% (95% CIs, 92.4–99.2) in the *TP53* mutation group vs. 97.3% (95% CIs, 94.1–98.8) in the *TP53* wild-type group (HR, 1.06; 95% CIs, 0.29–3.86; $p = 0.936$). (C) The 5-year DMFS rates: 89.8% (95% CIs, 83.8–93.6) in the *TP53* mutation group vs. 95.3% (95% CIs, 92.8–97.0) in the *TP53* wild-type group; the 10-year DMFS rates: 88.1% (95% CIs, 91.7–92.4) in the *TP53* mutation group vs. 91.0% (95% CIs, 87.3–93.6) in the *TP53* wild-type group (HR, 1.54; 95% CIs, 0.87–2.71; $p = 0.135$).

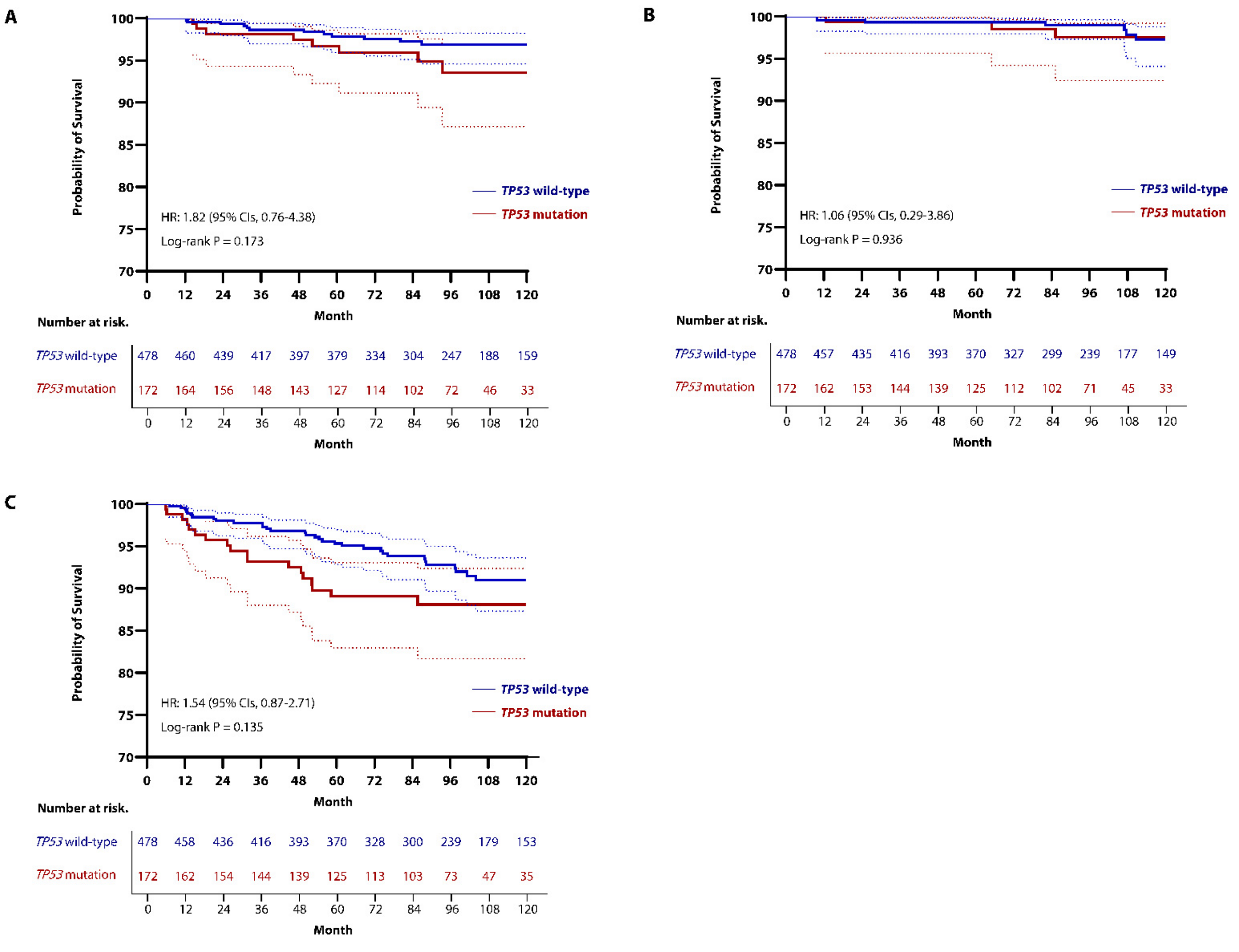

Figure S2. Kaplan-Meier curve for (A) LRFS, (B) RRFS, and (C) DMFS in patients with *TP53* mutation, stratified by types of mutation. Stratified log-rank test and Cox regression analysis presented that there were no significant differences between two groups. (A) The 5-year LRFS rates: 97.7% (95% CIs, 90.9–99.4) in the missense mutation group vs. 95.4% (95% CIs, 86.5–98.5) in the other mutations group; the 10-year LRFS rates: 97.7% (95% CIs, 90.9–99.4) in the missense mutation group vs. 88.1% (95% CIs, 74.5–94.7) in the other mutations group (HR, 0.24; 95% CIs, 0.05–1.19; *p* = 0.06). (B) The 5-year RRFS rates: 98.5% (95% CIs, 90.0–99.8) in the missense mutation group vs. 100% in the other mutations group; the 10-year RRFS rates: 98.5% (95% CIs, 90.0–99.8) in the missense mutation group vs. 96.9% (95% CIs, 88.0–99.2) in the other mutations group (HR, 1.43; 95% CIs, 0.13–15.72; *p* = 0.771). (C) The 5-year DMFS rates: 88.0% (95% CIs, 77.4–93.8) in the missense mutation group vs. 89.9% (95% CIs, 81.4–94.6) in the other mutations group; the 10-year DMFS rates: 88.0% (95% CIs, 77.4–93.8) in the missense mutation group vs. 88.2% (95% CIs, 78.9–93.5) in the other mutations group (HR, 1.13; 95% CIs, 0.45–2.87; *p* = 0.793)).

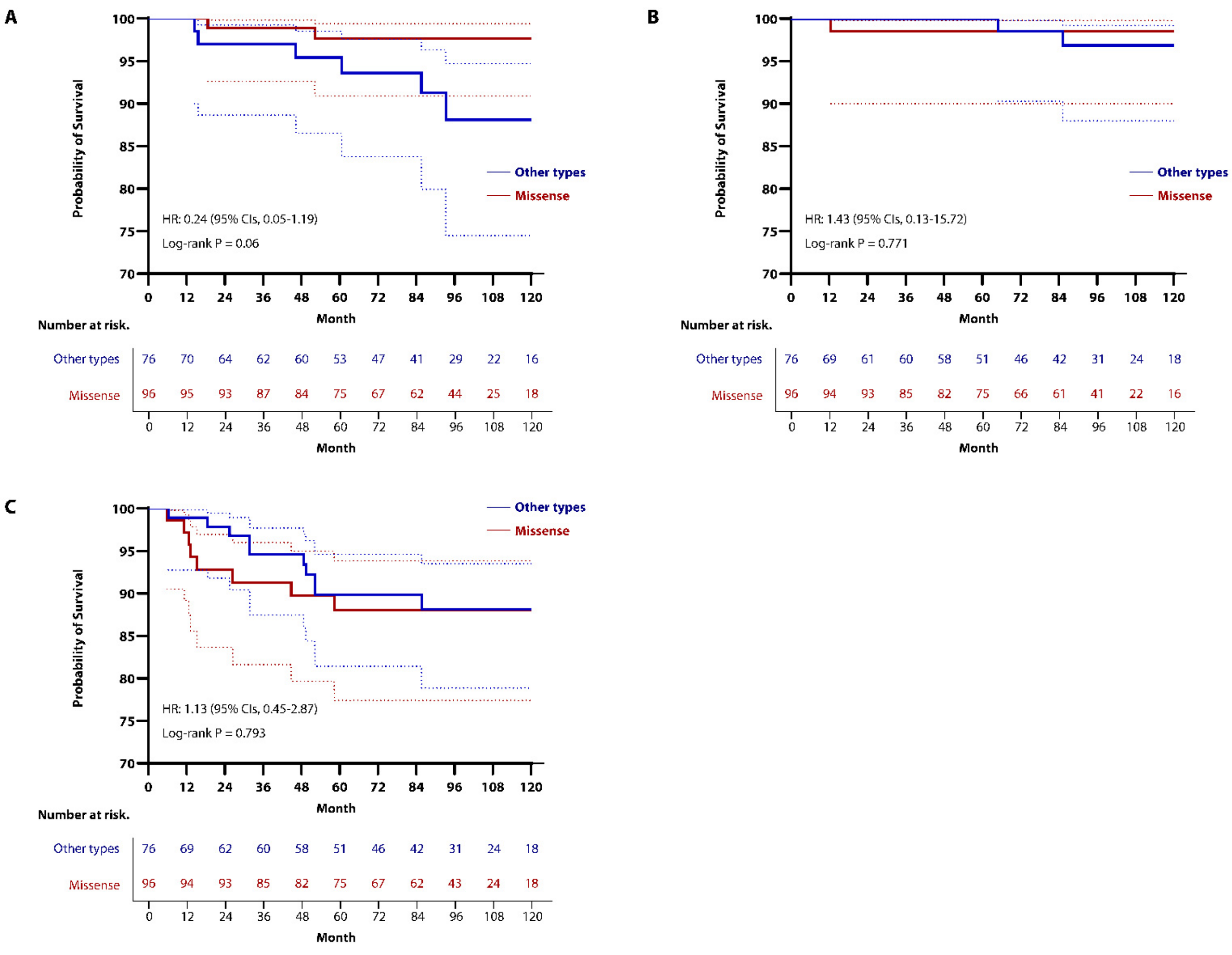

Figure S3. Kaplan-Meier curve for (A) LRFS, (B) RRFS, and (C) DMFS in patients with *TP53* mutation, stratified by locations of mutation. Stratified log-rank test and Cox regression analysis presented that there were no significant differences between two groups. (A) The 5-year LRFS rates: 96.9% (95% CIs, 92.0–98.8) in the DBD group vs. 95.0% (95% CIs, 69.5–99.3) in the other locations group; the 10-year LRFS rates: 93.3% (95% CIs, 86.0–96.9) in the DBD group vs. 95.0% (95% CIs, 69.5–99.3) in the other locations group (HR, 0.96; 95% CIs, 0.12–7.77; $p = 0.966$). (B) The 5-year RRFS rates: 99.3% (95% CIs, 95.1–99.9) in the DBD group vs. 100% in the other locations group; the 10-year RRFS rates: 97.2% (95% CIs, 91.4–99.1) in the DBD group vs. 100% in the other locations group (HR, 0.4; 95% CIs, 0–111526.0; $p = 0.509$). (C) The 5-year DMFS rates: 89.0% (95% CIs, 82.3–93.2) in the DBD group vs. 89.6% (95% CIs, 64.3–97.3) in the other locations group; the 10-year DMFS rates: 87.9% (95% CIs, 80.8–92.4) in the DBD group vs. 89.6% (95% CIs, 64.3–97.3) in the other locations group (HR, 0.84; 95% CIs, 0.19–3.65; $p = 0.814$).

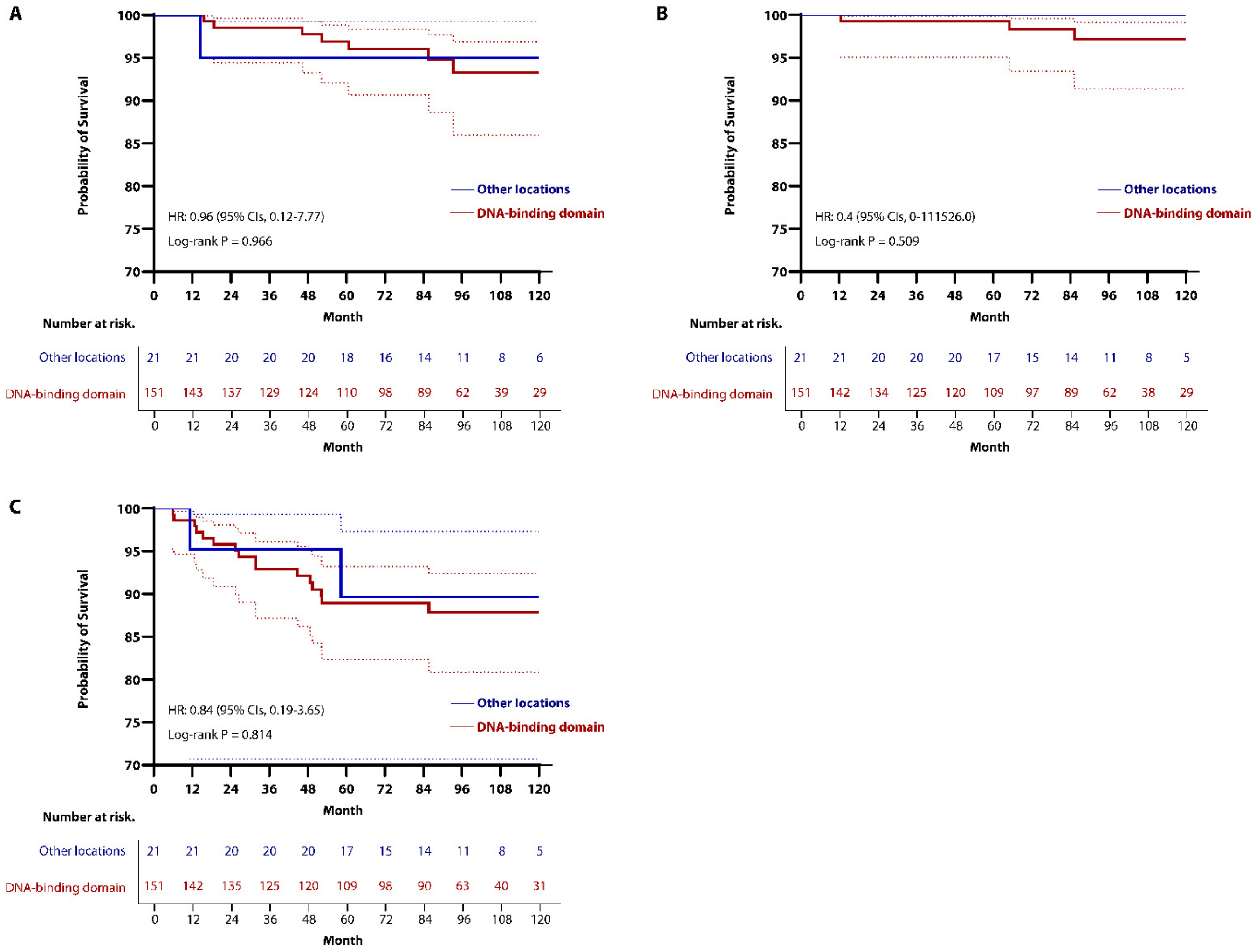

Figure S4. Kaplan-Meier curve for (A) LRFS, (B) RRFS, and (C) DMFS in patients with *TP53* mutation, stratified by the presence or absence of missense hotspot mutations. Stratified log-rank test and Cox regression analysis presented that there were no significant differences between two groups. (A) The 5-year LRFS rates: 100% in the missense hotspot mutations group vs. 95.9% (95% CIs, 90.4–98.3) in the other mutations group; the 10-year LRFS rates: 100% in the missense hotspot mutations group vs. 92.0% (95% CIs, 84.1–96.0) in the other mutations group (undefined HR, $p = 0.15$). (B) The 5-year RRFS rates: 100% in the missense hotspot mutations group vs. 99.2% (95% CIs, 94.6–99.9) in the other mutations group; the 10-year RRFS rates: 95.5% (95% CIs, 71.9–99.3) in the missense hotspot mutations group vs. 98.2% (95% CIs, 92.7–99.5) in the other mutations group (HR, 1.85; 95% CIs, 0.12–29.25; $p = 0.611$). (C) The 5-year DMFS rates: 100% in the missense hotspot mutations group vs. 86.3% (95% CIs, 78.9–91.3) in the other mutations group; the 10-year DMFS rates: 100% in the missense hotspot mutations group vs. 85.2% (95% CIs, 77.4–90.4) in the other mutations group (undefined HR, $p = 0.029$).

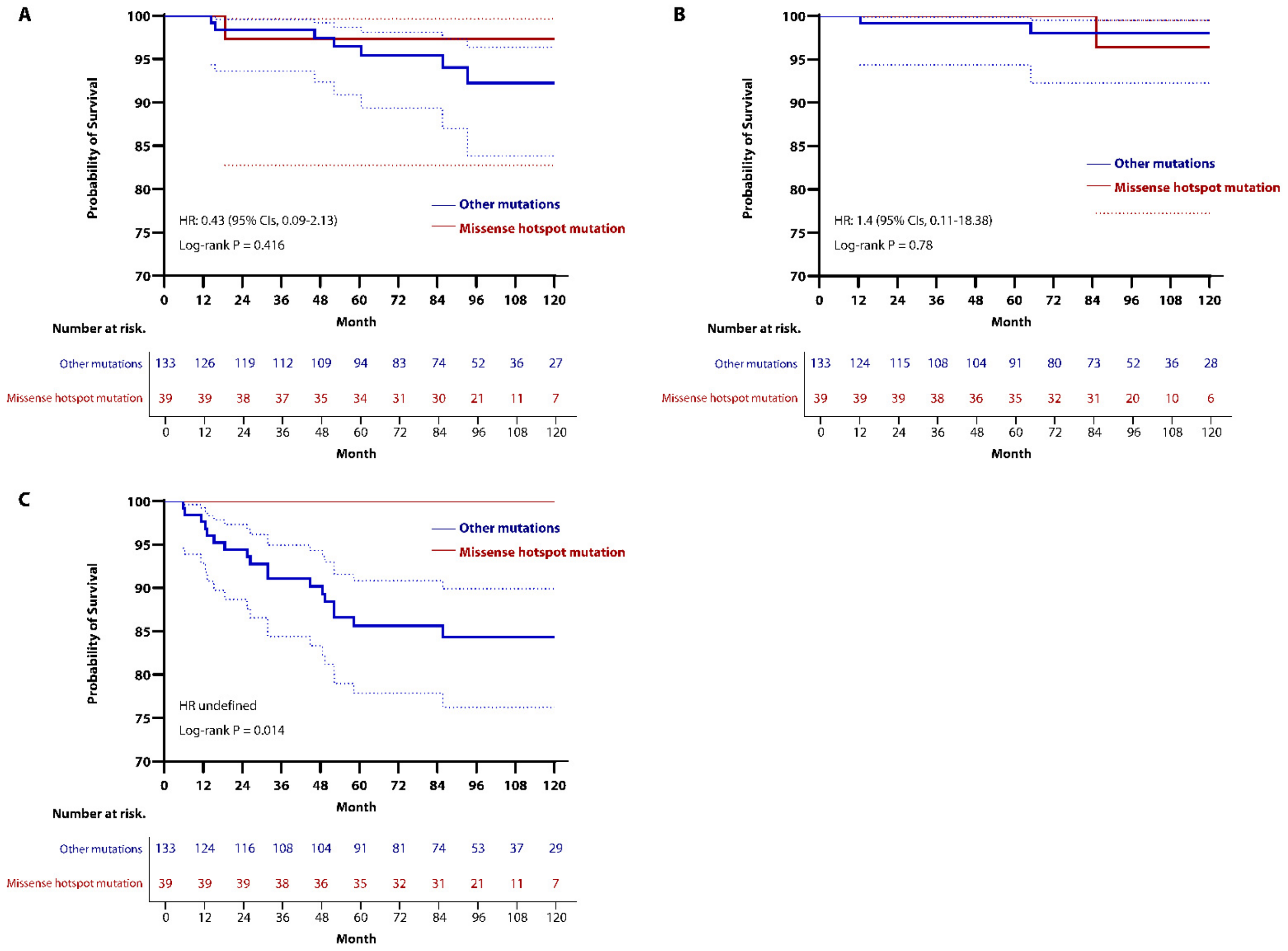

Figure S5. Kaplan-Meier curve for (A) RFS and (B) OS according to HER2-overexpression status within the *TP53*-mutated group. (A) The 5-year RFS rate in the HER2-positive subgroup was 91.3% (95% CIs, 81.6-96.0), and the 10-year RFS rate was 85.8% (95% CIs, 72.4-93.0), showing a trend better outcomes compared to the HER2-negative subgroup, which had a 5-year RFS rate of 83.1% (95% CIs, 73.4-89.4) and a 10-year RFS rate of 81.4% (95% CIs, 71.3-88.2), although the difference was not statistically significant (HR, 0.54; 95% CIs, 0.26-1.15; p = 0.137). (B) The 5-year OS rate was 94.1% (95% CIs, 85.1-97.8) in the HER2-positive subgroup and 93.0% (95% CIs, 85.0-96.8) in the HER2-negative subgroup, while the 10-year OS rate was 92.0% (95% CIs, 81.6-96.6) for the HER2-positive subgroup and 90.2% (95% CIs, 81.3-95.0) for the HER2-negative subgroup, with no statistically significant differences observed (HR, 0.81; 95% CIs, 0.27-2.43; p = 0.713).

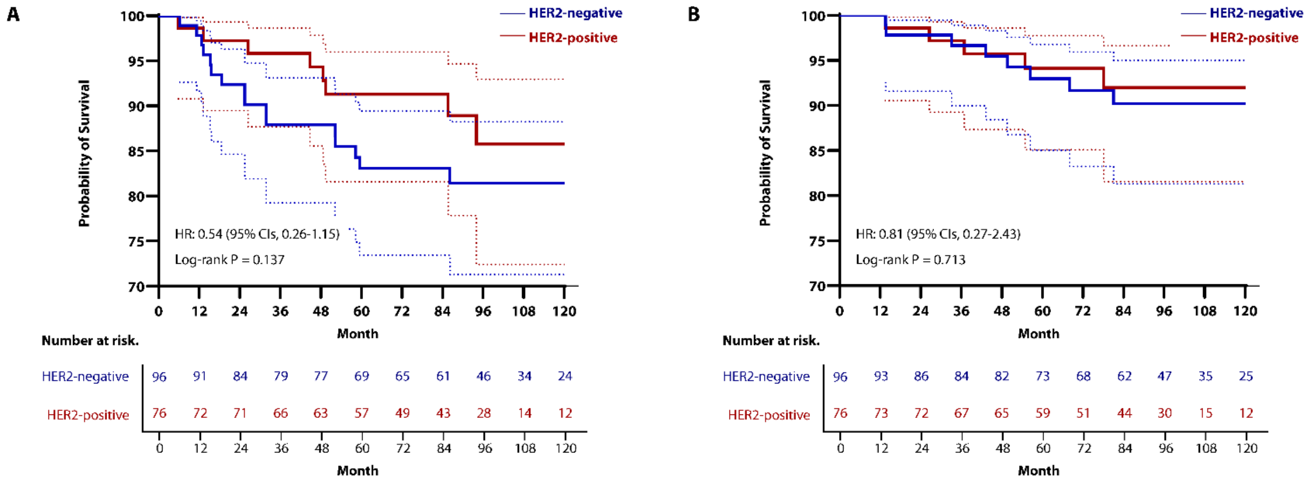

Supplement: Supplementary file 1 [file cancers-16-03899-s001.zip › cancers-3251842-supplementary.pdf]
